# Genetic diversity and inter-trait relationship of tropical extra-early maturing quality protein maize inbred lines under low soil nitrogen stress

**DOI:** 10.1371/journal.pone.0252506

**Published:** 2021-06-11

**Authors:** Pearl Abu, Baffour Badu-Apraku, Beatrice E. Ifie, Pangirayi Tongoona, Leander D. Melomey, Samuel K. Offei

**Affiliations:** 1 West Africa Centre for Crop Improvement, University of Ghana, Legon, Ghana; 2 International Institute of Tropical Agriculture (IITA), Ibadan, Nigeria; Kansas State University, UNITED STATES

## Abstract

Information on the genetic diversity, population structure, and trait associations of germplasm resources is crucial for predicting hybrid performance. The objective of this study was to dissect the genetic diversity and population structure of extra-early yellow and orange quality protein maize (QPM) inbred lines and identify secondary traits for indirect selection for enhanced grain yield under low-soil nitrogen (LN). One hundred and ten inbred lines were assessed under LN (30 kg ha ^-1^) and assayed for tryptophan content. The lines were genotyped using 2500 single nucleotide polymorphism (SNP) markers. Majority (85.4%) of the inbred lines exhibited wide pairwise genetic distances between 0.4801 and 0.600. Genetic distances were wider between yellow and orange endosperm lines and predicted high heterosis in crosses between parents of different endosperm colors. The unweighted pair group method with arithmetic mean (UPGMA) and the admixture model-based population structure method both grouped the lines into five clusters. The clustering was based on endosperm color, pedigree, and selection history but not on LN tolerance or tryptophan content. Genotype by trait biplot analysis revealed association of grain yield with plant height and ear height. TZEEQI 394 and TZEEIORQ 73A had high expressivity for these traits. Indirect selection for high grain yield among the inbred lines could be achieved using plant and ear heights as selection criteria. The wide genetic variability observed in this study suggested that the inbred lines could be important sources of beneficial alleles for LN breeding programs in SSA.

## Introduction

Information on the genetic diversity and population structure of inbred lines in hybrid breeding programs enables breeders to develop inbred lines with high heterosis in hybrid combinations and enhanced adaptation to specific or broad environmental conditions [1,2]. Knowledge of genetic diversity ensures efficient utilization of germplasm, which provides insurance against unexpected genetic drift and promotes sustainability of breeding programs [3]. Genetic distance information has been reliably used by maize breeders to resolve inbreds into heterotic groups and predict hybrid performance among tropical maize inbred lines [4]. Adu et al. [5] reported wide genetic distances among 94 early maturing white and yellow endosperm stress tolerant tropical maize inbred lines suggesting the presence of unique alleles for maize improvement. In another study by Abu et al. [6] significant correlation was observed between genetic distances and grain yield (GY) performance of tropical QPM maize inbred lines under low soil nitrogen (LN), *Striga* and optimal growing environments. Suwarno et al. [4] also reported correlations between single nucleotide polymorphism (SNP)-based heterotic groupings and high heterosis of selected provitamin A maize inbred lines. Oyekunle et al. [7] reported significant association of simple sequence repeat (SSR)-marker based heterotic grouping and genetic relationships with high heterosis under drought and optimal growing conditions.

Genetic diversity in maize populations can be determined using molecular, biochemical or morphological markers. However, molecular markers are the preferred choice because they are faster and more reliable for diversity studies and are independent of the environment, plant growth and developmetal stages [5]. DAlthough different types of markers such as SSR [8], amplified fragment length polymorphism (AFLP), restriction fragment length polymorphism (RFLP) [9,10] and single nucleotide polymorphism (SNP) [11] are available for maize diversity studies. However, SNP [10] markers are the preferred choice because they are more abundant in the genome, locus specific, cheaper and less prone to genotyping errors [11,12]. Several studies have reported significant genetic diversity within tropical maize germplasms and populations. For example, Adu et al. [14] reported wide genetic distances in 94 early maturing white and yellow stress tolerant endosperm maize inbred lines developed by the International Institute of Tropical Agriculture (IITA). The authors concluded that unique alleles were present in the inbred lines which could be utilized for genetic enhancement of maize in the tropics. Abu et al. [15] reported significant correlation between genetic distances and grain yield (GY) performance of tropical QPM maize inbred lines under low soil nitrogen (LN), *Striga* and optimal growing environments. Similarly, [8] reported significant correlations between SNP-based heterotic groupings and high heterosis of selected CIMMYT (International Maize and Wheat Improvement Centre) provitamin A maize inbred lines. Using SSR-markers [16], identified 42 early maturing tropical maize inbred parents for development of commercial hybrids with high heterosis under drought and optimal growing conditions.

The present global challenge to increase food production while protecting the environment neccesitates the the development of nitrogen use efficienct genotypes. In Africa, particularly sub-saharan Africa (SSA), however, poor soil fertility especially low soil nitrogen (LN) remains a widespread constraint to maize production and productivity [13]. Additionally, the SSA region has the lowest fertilizer application rate of about 15 kg ha^-1^ compared to the global average of 140 kg ha^-1^ [14]. Consequently, yield losses due to LN in SSA is estimated at over 50% [15]. Moreover, farmers in the region presently plant low yielding varieties with low quality protein content due to low lysine (1.5–2.5%) and tryptophan (0.025–0.050%) contents [16]. Quality protein maize (QPM) genotypes have optimum lysine and tryptophan contents and provide about 73% of the daily protein requirement compared to the 46% in normal endosperm maize [17]. Therefore, the development of LN tolerant QPM hybrids and open-pollinated varieties is critical to the successful promotion of soil health, increased grain yield and improved nutrition among resource poor farmers.

IITA, through its breeding efforts over the years has developed extra-early (80–85 days maturity) yellow and orange inbred lines from two *Striga* tolerant populations, 99 TZEE-Y Pop STR QPM and 2009 TZEE-OR2 STR QPM. To maximize the utilization of the germplasm for breeding LN tolerant QPM hybrids, it is imperative to determine the genetic diversity, population structure and important agronomic traits for selecting LN tolerant genotypes. Under the IITA- MIP, selection of *Striga* resistant/tolerant genotypes is carried out under low-N stress (30 kg N ha^-1^) conditions and has resulted in concomitant improvement of LN tolerance [18]. Therefore, it is anticipated that the newly developed yellow and orange QPM inbred lines would possess desirable alleles for LN tolerance since they were extracted from the *Striga* resistant/tolerant populations.

In breeding for LN tolerance, although high GY is the primary goal, secondary traits have been invaluable for indirect selection for high GY due to the low heritability of GY under stress. Therefore, secondary traits such as ears per plant, stay-green characteristic, anthesis-silking interval [19], plant height, ear height and plant aspect [20] have been employed by breeders for selecting for improved grain yield under low-N. Nonetheless, the reliability of secondary traits for indirect selection and hybrid performance prediction depends on the identification of highly heritable traits and genotypes with high expressivity for such traits [20]. Additionally, inter-trait relationship analysis such as genotype by trait (GT) biplot are efficient tools for correlation and trait profile analyses for multiple trait selection [21]. The tool is also effective for identifying reliable traits for indirect selection and the identification of the most suitable genotypes as parents for hybrid production [20]. The objectives of this study were to: (i). determine the genetic diversity and the population structure of tropical extra-early yellow and orange QPM inbred lines using SNP markers located within LN tolerance genomic regions, and (ii) dissect the inter-trait relationships among the inbred lines and identify the appropriate traits for selecting LN tolerant genotypes.

## Materials and methods

### Description of genetic materials

A total of 110 extra-early QPM inbred lines recently developed under the IITA- MIP were used (S1 Table). These included 53 yellow QPM inbred lines from population 99 TZEE-Y Pop STR QPM (designated TZEEQI), 53 orange QPM lines with favourable alleles for provitamin A derived from population 2009 TZEE-OR2 STR QPM (designated TZEEIORQ) and four white endosperm lines (designated TZEEQI-CHK) used as checks. Two of the checks (TZEEQI 7 and TZEEQI 60) are LN tolerant while the other two (TZEEQI 16 and TZEEQI 110) are susceptible based on the results of previous field studies. White endosperm lines were used as checks because no extra-early yellow or orange QPM checks were available at the time of the experiment.

### Assessment of inbred lines under low-soil nitrogen

Two test locations (Fumesua and Legon) in Ghana were used for the study during the minor planting season (September to November) of 2017. The first testing site was the Crop Research Institute-Fumesua (6°41′ N lat., 1°28′ W long.), with an altitude of 286 m above sea level, a semi-deciduous agro-ecological zone with annual rainfall of 1500 mm and temperatures of 21 ^o^C to 31 ^o^C. The second test site was the West Africa Centre for Crop Improvement (WACCI) Research Field-Legon (5°38′ N lat., 00^o^11′ E long.), with an altitude of 97 m above sea level, a Coastal Savanna agro-ecological zone with annual rainfall of 809 mm and temperatures of 23 ^o^C to 31 ^o^C. The Legon test site was depleted of N by planting maize at very high population density and removing the biomass at harvest. The Fumesua test site required no initial depletion because it had been previously depleted of N and used for LN trials only. Soil samples from each location were analyzed with Kjeldahl digestion calorimetric procedure [22] to ensure low N levels before planting. The nitrogen content of the soils at Legon and Fumesua were 0.06% and 0.11% respectively, which were considered lower than 0.2% minimum limit based on the Landon [23] interpretation (S2 Table).

The inbred lines were planted using a 10 x 11 alpha lattice design with two replications at each test location. Plot size was 4 m long single rows, with planting distance of 0.75 x 0.4 m. Three seeds were initially planted and thinned to two seeds per hill at 2 weeks after planting, resulting in population density of about 66,666 plants per hectare. Each plot received 15 kg ha^-1^ N, applied as urea (46% N) at two weeks after planting. Potassium (muriate of potash, 60% K) and phosphorus (triple super phosphate, 46% P) were applied at 60 kg ha^-1^ each. Top-dressing was carried out using 15 kg N ha^-1^ at 4 weeks after planting, bringing the total N to 30 kg N ha^-1^. Hand weeding was done to control weeds when necessary.

### Determination of the tryptophan content of the inbreds

Seed samples of 65 (55 yellow plus 10 orange lines) selected inbred lines were dispatched to the Maize Nutritional Quality and Plant Tissue Analysis Laboratory of CIMMYT, for tryptophan analysis using the calorimetric method [24]. The 65 inbred lines were selected because they were newly developed lines with high levels of pro-vitamin A and/or lysine and tryptophan contents, and were also of major interest for the combining ability studies [6]. Tryptophan content of each genotype was estimated from two independent samples. Although lysine and tryptophan are equally important determinants of the protein content in maize, only tryptophan was analyzed due to high cost implications. Furthermore, the correlation between tryptophan and lysine is positive and very high [24].

### Data collection

Days to anthesis (DA) was recorded as number of days from planting to when 50% of the plants in a plot had shed pollen. Days to silking (DS) was obtained as the number of days from planting when 50% of the plants in a plot had silks. Anthesis silking interval (ASI) was the difference between DS and DA. The stay green characteristic was scored at 70 days after planting (DAP) based on leaf senescence using a scale on 1–9; where 1 = 0–10% dead leaf area and 9 = 91–100% dead leaf area, respectively. Plant aspect was scored on a scale of 1–9; where 1 = excellent plant phenotypic appearance and 9 = poor plant phenotypic appearance [25]. Plant and ear heights were the mean of five random plants measured as the distance from the ground to the first tassel branch and the node bearing the upper ear, respectively. Husk cover was scored on a scale of 1–5; where 1 = tightly arranged husks extending beyond the tip of the ear and 5 = completely exposed ear tip. The number of ears per plant was calculated as number of ears harvested per plot divided by the number of plants harvested. Ear aspect was scored on a scale of 1–9; where 1 = uniform, well-filled, large and disease-free ears and 9 = undesirable ear features [25]. Grain yield (kg ha^-1^) in each plot was calculated using the grain weight and moisture content of shelled ears and 15% adjusted moisture content.

### Genotyping of the inbred lines with single nucleotide polymorphism (SNP) markers

Out of the 110 inbred lines studied, 103 were genotyped because seven lines did not germinate in the nursery. Leaf samples were taken from two-week-old plants and dispatched to LGC Company (http://www.lgcgroup.com/) for DNA extraction and genotyping. The targeted genotyping by sequencing service (SeqSNP) platform provided by LGC genomics was used (https://www.biosearchtech.com/services/sequencing/targeted-genotyping-by-sequencing-seqsnp). A total of 2500 SNP markers were selected from the 50 K maize SNP genotyping array by [26] to genotype the inbred lines. The markers were selected based on QTL and genomic regions of LN tolerance traits identified in previous studies with SNP [27–32] or SSR [33,34] markers. For studies that used SSR markers, the physical positions of the QTLs were obtained from the Maize Genetics and Genomics Data base, MaizeGDB [35], (https://www.maizegdb.org/) to enable selection of SNPs targeting the relevant QTLs. The schematic map of the QTLs used for the selection of SNPs for this study can be accessed from the cited references.

### Data analysis

#### Phenotypic data analysis

Analysis of variance (ANOVA) was performed using the general linear model procedure (PROC GLM) of the Statistical Analysis System (SAS) software adopting a random statement with test option [36]. Each location was considered as a test environment. Replications and environments were treated as random factors and inbred lines as fixed factors. The entry means were adjusted for block effects according to the lattice design [37]. Means were separated with the least significant difference (LSD). The restricted maximum likelihood (REML) method was used to estimate the broad sense heritability for each trait using the phenotypic and genotypic variance components via PROC Varcomp in SAS.

The low nitrogen base index (LNBI) proposed by [20] that combined grain yield (GYLD), number of ears per plant (EPP), anthesis-silking interval (ASI), plant aspect (PA), ear aspect (EA) and the stay green characteristic (STYG) was used to identify LN tolerant genotypes;

LNBI=(2xGYLD)+EPP–ASI–PA–EA–STYG

Prior to the analysis, all traits were standardized using a standard deviation of 1 and mean of zero to reduce the effects of the different scales used for measuring the various traits. Therefore, a positive LNBI value indicated LN tolerance and the negative value susceptibility.

Genotype-by trait biplot analysis was done using the standardized means of the measured traits and GGE biplot procedure [38] in the R software version 3.6.1. The analysis was performed by installing the GGE Biplot GUI and GGE Biplot packages in R (http://www.rproject.org/).

#### Genotypic data analysis

The data was filtered in TASSEL version 5.2.53 [39] to retain only markers with missing data <10% and minor allele frequency (MAF) > 5%. This resulted in 1691 markers for the diversity study. Gene diversity, heterozygosity, major allele frequency and polymorphic information content (PIC) were estimated in PowerMarker version 3.25 [40]. Nei’s frequency based genetic distances [41], the unweighted pair group method with arithmetic mean (UPGMA) and 1000 nonparametric bootstrapping across different loci were used for the cluster analysis in PowerMarker. Phylogenic tree was viewed in MEGA X software [42]. Population structure analysis was done using the admixture model-based method of the STRUCTURE software version 2.3.4 [43]. In this model, K represented the number of clusters which were initially fixed from 1 to 12 with 10 alterations per each K and run against 10,000 Markov Chain Monte Carlo (MCMC) with 10,000 burn-in. The best K for grouping the inbred lines into clusters was determined by importing the output from the structure analysis into the STRUCTURE HARVESTER [44]. A threshold of 70% was used to classify each inbred line into a cluster and the inbred lines below threshold were classified into the admixture cluster [45,46].

## Results

### Genotypic variation and mean performance of inbred lines under low soil nitrogen

Combined analysis of variance (ANOVA) revealed significant (P<0.01) differences among the inbred lines for all measured traits across the two LN environments (Table 1). Genotype by environment interaction (G X E) was significant for all traits except days to silking (DS) and ear aspect (EA). Broad sense heritability estimates were low to moderately high for measured traits, ranging between 15% for ears per plant (EPP) and 85% for DS. Broad sense heritability for grain yield (GY) was 51%. TZEEIORQ 73A was the only inbred line that out-yielded the check TZEEQI 60 (Table 2). The inbreds displayed varying levels of tolerance to LN, high yield, and EPP, shorter ASI, lower scores for plant aspect (PA) and the stay-green characteristic (STYG). Of the 110 lines, 50.1% (56 lines) had positive LN base index scores. The GY of 37 inbred lines were higher than the mean GY. The performance of the orange lines was slightly better than that of the yellow lines based on the index scores. About 52.8% and 49.1% of the orange and yellow lines had positive base indices, respectively. The mean index scores of the orange and yellow lines were 0.2 and -0.2, respectively. The tryptophan contents of the selected lines ranged from 0.044% for TZEEQI 357 to 0.094 for TZEEQI 414 with a mean of 0.064% (S3 Table). Of the 65 inbred lines selected based on the tryptophan contents, 25 satisfied the minimum level of 0.07% [27], with 14 of having positive base indices. Four inbred lines, TZEEIORQ 73A, TZEEQI 392, TZEEQI 394 and TZEEQI 408 displayed LN tolerance and had optimal tryptophan contents of 0.08%, 0.07%, 0.07% and 0.08%, respectively. Fifteen inbred lines displayed positive base index values but had sub-optimal tryptophan contents (S3 Table).

**Table 1:** Mean squares and heritability estimates of the inbred lines under LN stress.

Source	DF	GY (kg ha-^1^)	DA	DS	ASI	STYG (1–9)	PA (1–9)	EA (1–9)	EPP	HC (1–5)	PHT (cm)	EHT (cm)
Block (Env*Rep)	40	158288 (p = 0.022)	9.7 (p<0.001)	10.2 (p<0.001)	1.5 (p = 0.008)	0.39ns	0.73 (p = 0.036)	0.29ns	0.08 (p = 0.049)	0.89ns	243.6 (p = 0.009)	87 (p = 0.004)
Rep (Env)	2	2891037 (p<0.001)	32.5 (p<0.001)	35.4 (p<0.001)	1.8ns	0.85ns	0.82ns	2.00 (p = 0.002)	0.39 (p<0.001)	2.97 (p = 0.013)	3571.4 (p<0.001)	1230 (p<0.001)
Genotype	109	297090 (p<0.001)	29.5 (p<0.001)	29.5 (p<0.001)	2.8 (p<0.001)	0.84 (p<0.001)	0.85 (p<0.001)	0.79 (p<0.001)	0.12 (p<0.001)	2.68 (p<0.001)	741.8 (p<0.001)	232 (p<0.001)
Env	1	9002574 (p<0.001)	2539.2 (p<0.001)	189.8 (p<0.001)	1340.5 (p<0.001)	14.18 (p<0.001)	129.82 (p<0.001)	8.52 (p<0.001)	16.69 (p<0.001)	25.06 (p<0.001)	256040 (p<0.001)	37116 (p<0.001)
Genotype* Env	109	149315 (p = 0.008)	5.4 (p = 0.002)	4.82ns	2.1 (p<0.001)	0.48 (p = 0.037)	0.72 (p = 0.006)	0.39ns	0.11(p<0.001)	1.11 (p<0.001)	203.57 (p = 0.016)	84.28 (p<0.001)
Error	178	99514	3.3	4	0.9	0.36	0.47	0.3	0.05	0.66	141.84	47.79
Heritability		0.51	0.84	0.85	0.28	0.45	0.17	0.52	0.15	0.61	0.74	0.67

ns = not significant, Env = environment, Rep = replication, GY = grain yield, DA = days to 50% anthesis, DS = days to 50% silking, ASI = anthesis-silking interval, STYG = stay-green characteristic, EA = ear aspect, EPP = ears per plant, HC = husk cover, PHT = plant height, EHT = ear height.

**Table 2:** Grain yield and other traits of 25 (top 15 and worst 10) inbred lines along with four checks under LN stress conditions.

INBRED	GY (kg ha^-1^)	DA	DS	ASI	STYG (1–9)	PA (1–9)	HC (1–5)	PHT (cm)	EHT (cm)	EA (1–9)	EPP	LNBI
TZEEQI 394	1313.83	55.92	56.97	1.04	5.12	4.32	1.58	128.34	51.61	4.12	1.20	9.30
TZEEIORQ 73A	1943.55	50.38	51.96	1.58	5.44	4.63	3.44	130.12	66.71	4.84	0.91	8.50
TZEEIORQ 22A	1484.05	57.57	58.76	1.18	4.73	5.41	1.88	123.61	62.87	4.20	1.09	8.19
TZEEIORQ 4	1344.59	58.91	61.07	2.16	4.69	4.17	1.44	119.89	63.44	4.61	1.05	7.51
TZEEQI 408	1117.07	57.29	58.43	1.14	3.91	4.37	3.87	111.45	60.00	4.90	0.97	7.35
TZEEIORQ 17	1554.68	56.34	57.28	0.94	4.56	5.29	2.54	119.59	58.18	5.18	1.04	7.16
TZEEIORQ 5	1056.81	54.67	55.47	0.79	4.37	3.82	4.08	120.04	57.99	5.62	1.18	7.08
TZEEIORQ 63A	857.65	55.26	56.97	1.71	4.01	3.91	2.98	135.57	52.15	5.06	1.21	6.65
TZEEQI 392	1392.37	55.31	57.10	1.79	4.78	4.17	2.31	126.08	62.43	4.84	0.84	6.48
TZEEQI 7-Check 1	772.14	55.69	56.67	0.98	4.49	4.65	2.11	106.33	41.71	5.03	1.44	5.81
TZEEQI 357	906.23	55.06	56.28	1.22	4.24	4.23	3.54	95.63	41.54	4.42	0.69	5.09
TZEEIORQ 61A	1023.04	52.29	54.10	1.80	4.16	4.72	2.37	125.00	45.05	5.04	1.06	5.01
TZEEIORQ 72A	977.85	58.79	60.77	1.99	4.41	4.71	3.77	128.17	42.45	5.15	1.23	4.69
TZEEQI 393	941.83	56.01	57.03	1.03	4.78	4.80	3.31	123.11	61.22	4.78	1.03	4.34
TZEEIORQ 34	787.26	58.35	59.68	1.32	4.29	4.94	1.82	121.71	50.80	4.55	1.03	4.25
TZEEIORQ 3	808.03	59.64	60.42	0.77	4.46	5.20	2.35	106.34	52.38	4.88	1.13	4.01
TZEEQI 60-Check 2	1204.08	56.21	58.46	2.25	4.54	4.61	2.10	131.59	53.34	4.86	0.77	3.94
TZEEQI 110-Check 3	380.90	57.01	60.15	3.14	4.14	4.31	3.00	87.61	36.52	5.44	0.76	-2.31
TZEEQI 368	21.67	60.86	62.65	1.80	4.04	4.64	4.85	82.77	35.22	6.12	0.72	-5.21
TZEEQI 16-Check 4	360.17	59.60	62.57	2.96	5.20	4.55	2.36	130.95	67.83	5.19	0.57	-5.34
TZEEQI 359	407.27	55.15	57.50	2.35	5.71	5.14	2.54	115.16	52.19	4.98	0.64	-5.69
TZEEIORQ 67	369.64	58.81	60.01	1.20	5.42	5.52	3.06	102.61	49.86	4.97	0.35	-6.27
TZEEIORQ 13A	562.72	54.71	58.03	3.32	5.22	6.54	2.33	110.97	55.78	5.10	0.82	-6.73
TZEEIORQ 50B	551.14	52.96	56.46	3.50	5.04	5.36	4.85	114.14	55.88	5.86	0.61	-7.16
TZEEQI 351	528.22	51.29	53.80	2.50	5.82	6.36	3.59	97.43	45.87	4.87	0.49	-8.15
TZEEQI 411	197.49	53.03	55.95	2.92	3.21	4.97	3.81	102.95	36.70	6.22	0.69	-8.71
TZEEIORQ 50	395.36	53.98	57.02	3.03	5.06	6.03	4.65	102.39	51.43	5.95	0.64	-8.98
TZEEQI 363	314.95	55.78	59.92	4.15	4.82	4.84	2.79	100.33	36.95	6.33	0.51	-9.51
TZEEIORQ 6A	233.19	53.31	54.87	1.56	5.34	6.02	4.47	91.38	44.41	7.04	0.77	-10.51
**Mean**	**684.26**	**56.11**	**58.23**	**2.13**	**4.70**	**4.87**	**2.88**	**114.76**	**51.23**	**3.1481**	**0.91**	
**LSD**	**440.19**	**2.53**	**2.79**	**1.29**	**0.83**	**0.96**	**1.14**	**16.62**	**9.65**	**0.76**	**0.32**	

CHK = check, GY = grain yield, DA = days to anthesis, DS = days to silking, ASI = anthesis-silking interval, STYG = stay-green characteristic, PA = plant aspect, HC = husk cover, PHT = plant height, EHT = ear height, EA = ear Aspect, EPP = ears per plant, LNBI = low nitrogen base index.

### Inter-trait relationship and correlation among traits

**Fig 1:**
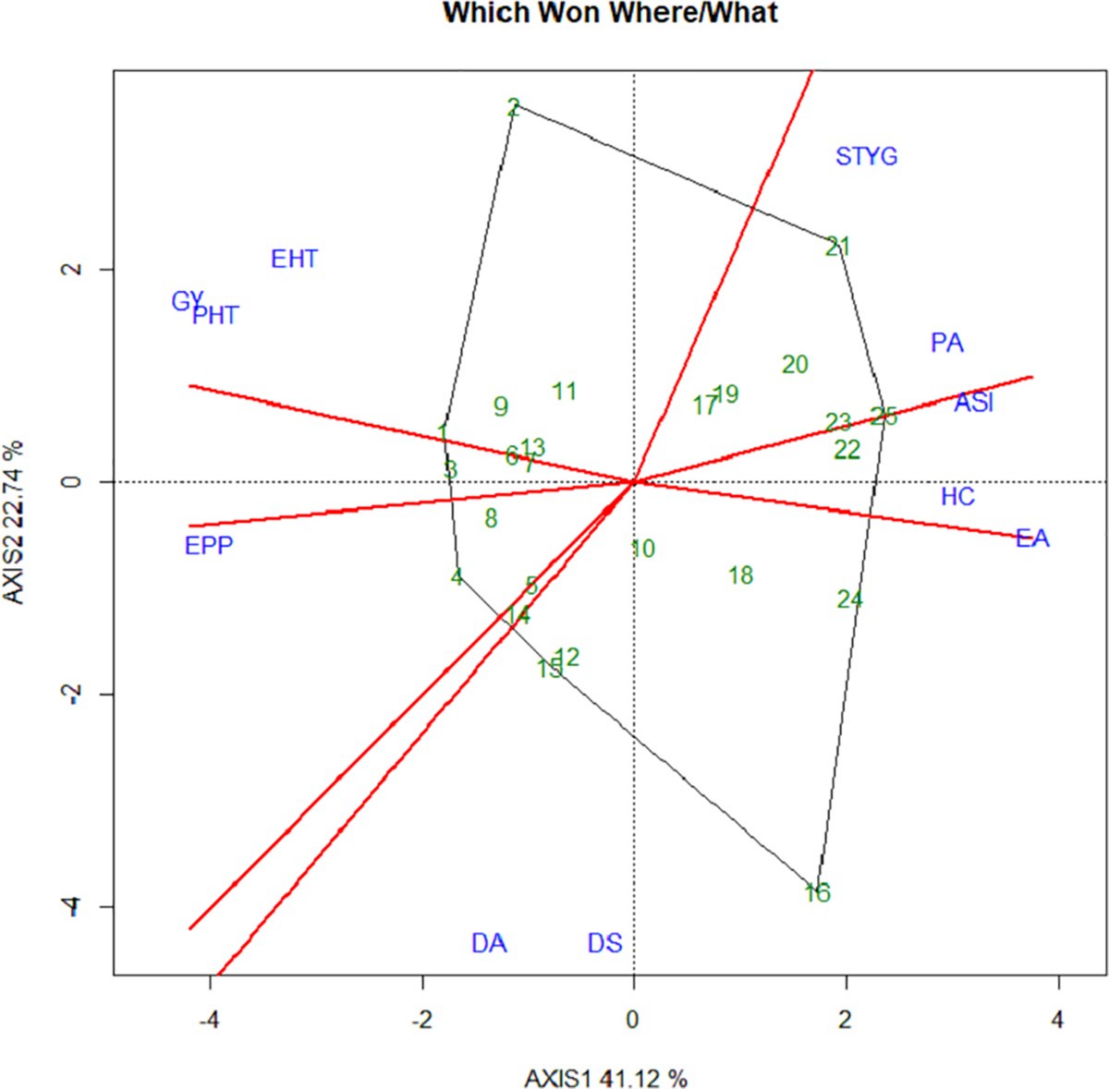
A ‘which won where/what genotype-by-trait biplot view of 25 (15 best and 10 worst) selected inbred lines evaluated under LN stress. GY = grain yield (kg ha^-1^), DA = days to 50% anthesis, DS = days to silking, ASI = anthesis-silking interval, STYG = stay-green characteristic, PA = plant aspect, PHT = plant height, EHT = ear height, HC = husk cover EA = ear Aspect, EPP = ears per plant. 1 = TZEEQI 394; 2 = TZEEIORQ 73A; 3 = TZEEIORQ 22A; 4 = TZEEIORQ 4; 5 = TZEEQI 408; 6 = TZEEIORQ 17; 7 = TZEEIORQ 5; 8 = TZEEIORQ 63A; 9 = TZEEQI 392; 10 = TZEEQI 357; 11 = TZEEIORQ 61A; 12 = TZEEIORQ 72A; 13 = TZEEQI 393; 14 = TZEEIORQ 34; 15 = TZEEIORQ 3; 16 = TZEEQI 368; 17 = TZEEQI 359; 18 = TZEEIORQ 67; 19 = TZEEIORQ 13A; 20 = TZEEIORQ 50B; 21 = TZEEQI 351; 22 = TZEEQI 411; 23 = TZEEIORQ 50; 24 = TZEEQI 363; 25 = TZEEIORQ 6A.

The genotype-by-trait biplot (GT) analysis was performed to study the inter-relationships among the measured LN tolerance traits and to select parental lines and predict hybrid performance (Fig 1). The top 15 best and worst 10 performing lines were selected using the GT biplot analysis and the base index scores. The first and second principal component axis (PC1 & PC2) explained 63.86% of the total variation among the lines. In the biplot polygon view, high values of GY, PHT, EHT and EPP were desirable while high values for DA, DS, ASI, STYG, PA, EA and husk cover (HC) were undesirable. Genotypes located at the vertex of the polygon were the winning genotypes for the traits located within that sector. The distance of a genotype from the biplot origin represented the uniqueness of the genotype relative to the mean for all genotypes. Thus genotypes that had average performance across the traits were positioned closer to the biplot origin whereas genotypes that performed above average across the traits were positioned away from the origin. The results showed that inbred lines 1 (TZEEQI 394) and 2 (TZEEIORQ 73A) performed better in terms of GY, PHT and EHT while inbred line 4 (TZEEIORQ 4) had the highest number of EPP. In terms of flowering date, inbred 16 (TZEEQI 368) performed poorly (later maturity) due to the higher DA and DS values. Inbred lines 21 (TZEEQI 351) and 25 (TZEEIORQ 6A) were located at the vertex of the sector containing STYG and PA indicating poor performance of these traits resulting in poor GY.

### Summary statistics of SNP markers and genetic distance

**Fig 2:**
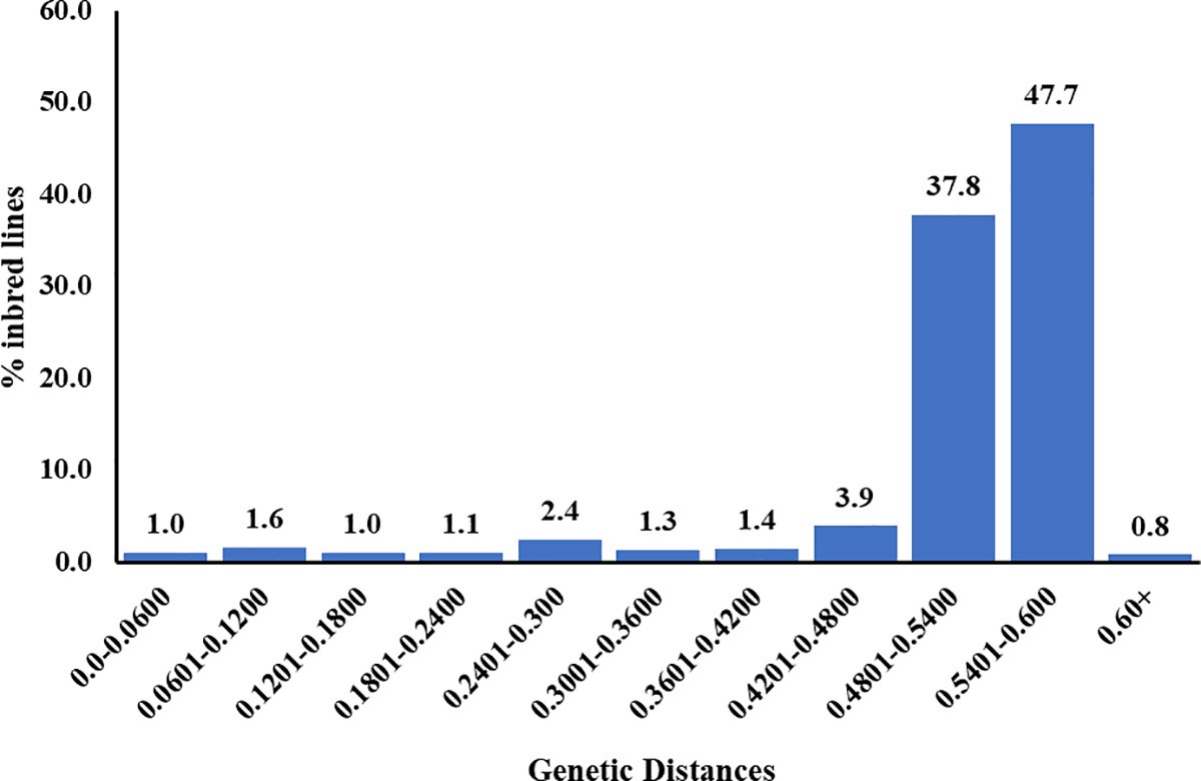
Frequency distribution of pairwise genetic distances among the inbred lines estimated using the Nei’s method using 1691 SNP markers.

The genetic diversity among the newly developed extra-early yellow and orange QPM inbred lines was studied using 1691 informative markers to obtain information for parent selection in hybrid breeding programs. Major allele frequency of the SNPs ranged from 0.500 to 0.990 with a mean of 0.725. (Table 3). Gene diversity varied from 0.019 to 0.607 with an average of 0.363. Polymorphic information content (PIC) ranged from 0.019 to 0.535 with a mean of 0.290. The average heterozygosity for the 1691 markers was 0.070 and ranged from 0.00 to 0.590. The mean pairwise genetic distance was 0.508 and varied between 0.0098 and 0.615 (Fig 2). Generally, the genetic distances were higher between yellow and orange lines. The lowest genetic distance was detected between TZEEIORQ 50 and TZEEIORQ 50B. These two inbred lines have orange endosperm, are related by descent, and shared similar selection history based on their pedigree information (S1 Table). The highest genetic distance was detected between TZEEQI 16 and TZEEIORQ 6A followed by TZEEQI 393 and TZEEIORQ 9A. These pairs of lines differed both in endosperm color and the source populations from which they were extracted. In general, most of the inbred lines had pairwise genetic distances between 0.4801 to 0.600, 37.7% and were between 0.4801–0.5400 and 47.7% and between 0.5401–0.600.

**Table 3:** Summary statistic of 1691 SNP markers used to study the genetic diversity.

Marker	MajAF	GenDiv	Het	PIC	MinAF
Minimum	0.500	0.019	0.000	0.019	0.010
Maximum	0.990	0.607	0.594	0.535	0.500
Mean	0.725	0.363	0.070	0.290	0.275

MajAF = major allele Frequency, GenDiv = gene diversity, Het = Heterozygosity, PIC = polymorphic information content, MinAF = minor allele frequency.

#### Cluster and population structure

**Fig 3:**
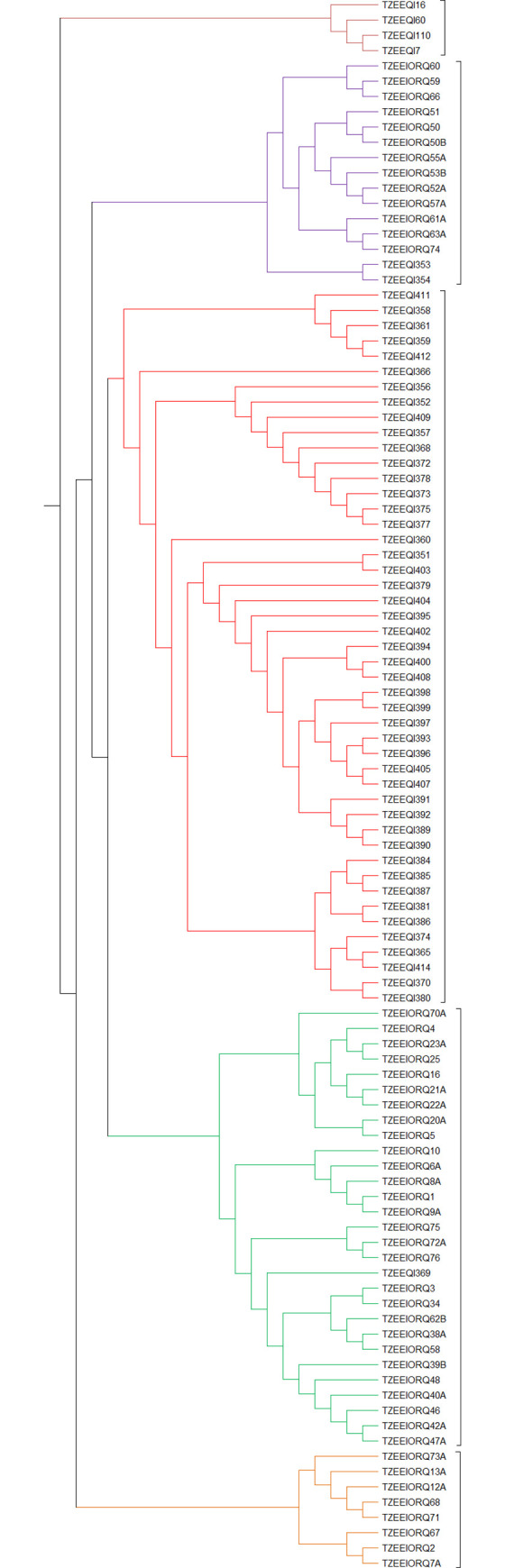
Clustering patterns of the inbred lines based on Nei’s genetic distances with 1691SNP markers using the UPGMA dendrogram method.

The UPGMA cluster analysis grouped the inbred lines into five main clusters using Nei’s genetic distances (Fig 3). Group 1 comprised four inbred lines, group 2, 3, 4, and 5 had 15, 47, 29 and 8 inbred lines, respectively. Groups 2, 3, 4 and 5 were further divided into two sub-groups. The extra-early white checks were distinctively classified into group 1. The remaining four groups contained either yellow or orange endosperm lines. Group 2 had orange lines that shared similar selection history with the two yellow lines, TZEEQI 353 and TZEEQI 354. Generally, the inbred lines clustered based on the source populations, endosperm colors, selection history and pedigrees. No distinct clustering pattern was detected based on LN tolerance or tryptophan content.

**Fig 4:**
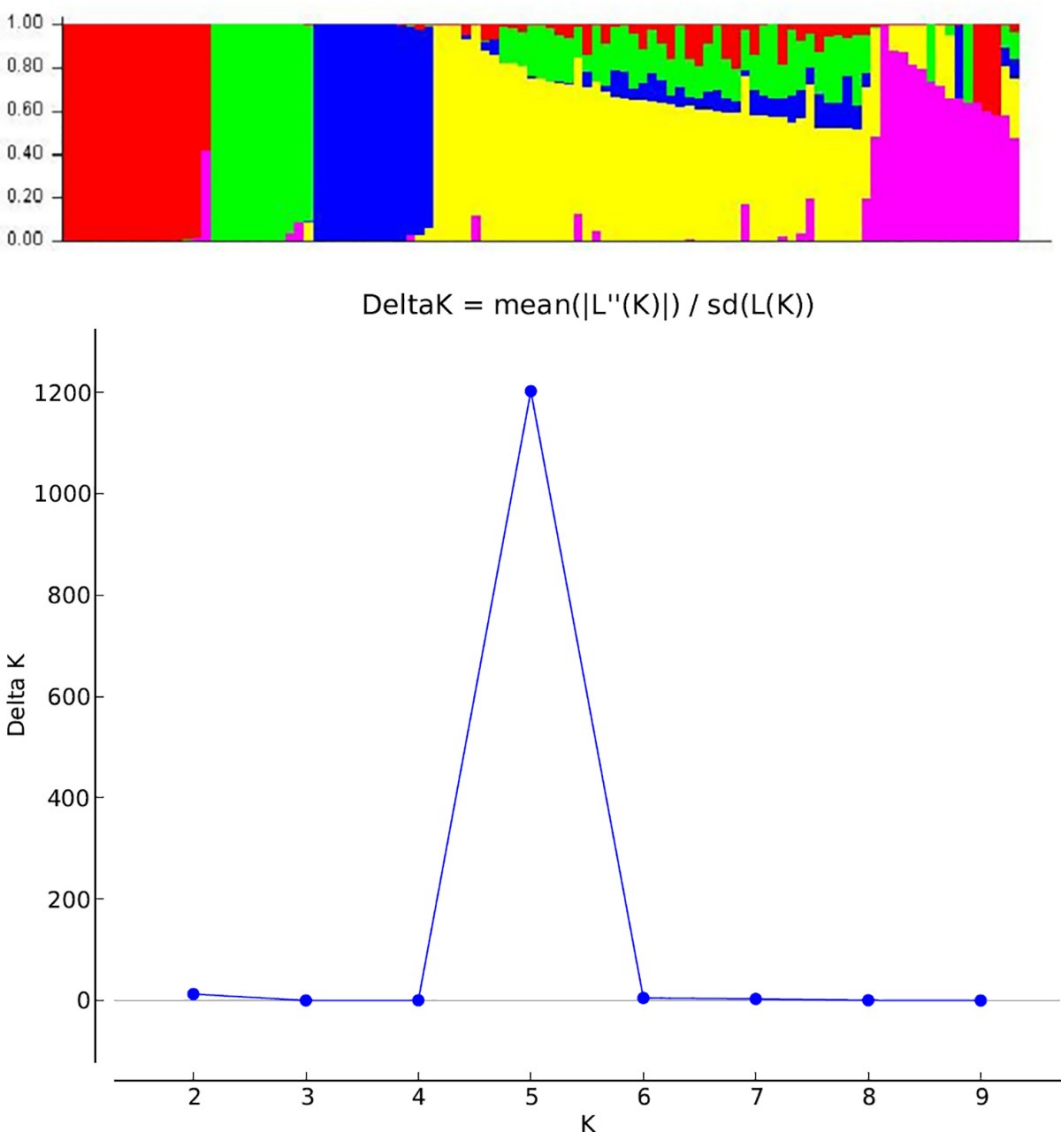
a. Population structure bar plot of the inbred lines showing five sub-populations of the 103 extra-early QPM/ Provitamin A inbred. b. A graph showing of the best K with the Evanno method used to group the inbred lines into sub-populations at k = 5.

In the population structure analysis, the inbred lines were grouped into five sub-populations (Fig 4A) based on the highest peak for the ad hoc K = 5 (Fig 4B). Although the population structure analysis revealed 5 sub-populations just as the UPGMA cluster analysis, the inbred lines in each of the five groups were quite different. However, all the 13 inbred lines in the first sub-group of the cluster analysis (Fig 3) were distinctively grouped into sub-population 3 (Fig 4A). In the population structure analysis, sub-populations 1, 2, 3, 4, and 5 had 15 (14.6%), 11 (10.7%), 13 (12.6%), 17 (16.5%), and 7(6.8%) lines, respectively (S1 Table). About 38.8% (40 lines) had probability of association below the 70% threshold and were classified into the mixed population group. Sub-populations 1 and 5 had only yellow endosperm lines, sub-population 2 and 3 had only orange endosperm lines while sub-population 4 had 71% yellow lines. All the white endosperm lines were classified into the mixed group. Generally, the groupings of the lines were based on endosperm color, selection history and pedigree information. No specific pattern of grouping was detected based on LN tolerance or tryptophan content. The population structure analysis estimated the fixation index (F_ST_) for each sub-population and indicated significant divergence between the five sub-populations. The F_ST_ values for populations 1–5 were 0.29, 0.51, 0.72, 0.43 and 0.41, respectively.

## Discussion

The development of high yielding QPM and LN tolerant varieties is critical for addressing the present malnutrition and food security challenges in sub-Saharan Africa (SSA). The success of a stress tolerant hybrid breeding program depends largely on the performance of the inbred parents and the heritability of traits under stress [47]. Understanding of the genetic diversity of inbred lines enables the selection of genetically divergent parents to maximize heterosis for hybrid production [1,3]. This study was conducted to access the genetic diversity and LN tolerance among a set of newly developed extra early yellow and orange endosperm QPM inbred lines in order to maximize the potential for hybrid production and population improvement in breeding programs in SSA. High genetic variation was detected for GY and other measured traits suggesting that selection could result in significant gains. Bänziger et al. [48]and Presterl et al. [49] reported the existence of significant genetic variation for LN tolerance in maize. The environmental effects were significant for GY and most traits and indicated significant climatic and edaphic differences in the two test locations. The significant genotype by environment effects suggested an inconsistency in the ranking of performance of the lines across the two locations. This is attributable to the divergent environmental factors prevailing in the two locations probably due to the differences in temperature, soil types, amount of rainfall and irrigation systems in the two locations. The experiments for both locations were conducted during the minor planting season (September–November) under rain-fed conditions supplemented with sprinkler or drip irrigation. Annor and Badu-Apraku [50] also reported significant G X E under similar environments. This underpins the significance of the environment in identifying LN tolerant genotypes and the need to test parents across contrasting locations and identify the best parents for specific locations [51]. The heritability estimates for GY and most traits were moderate to high, suggesting that a large proportion of the genetic variability was due to genes rather than the environment. This implied that direct phenotypic selection for the measured traits might have resulted in genetic gains. Heritability estimates were however low for ASI and EPP which are both secondary traits employed in the base index for selection for LN tolerance. Bänziger et al. [48] and Badu-Apraku et al. [20] had suggested that ASI and EPP to be highly heritable traits for selection for high GY under LN and thus included the trait in their proposed selection index.

Fifty-six out of the 110 inbred lines studied displayed positive LN base indices although the populations were not improved for LN tolerance. This is attributable to the screening method adopted to improve the populations for *Striga* tolerance that uses low N levels (30 kg ha^-1^), and probably induced concomitant improvement for LN tolerance. The result corroborated the previous findings of [18] who reported outstanding performance of *Striga* tolerant lines under LN and suggested that selection for *Striga* resistance under LN resulted in simultaneous improvement in LN tolerance. The degree of LN tolerance was higher for the orange lines compared to the yellow line suggesting that the orange lines had undergone more cycles of genetic enhancement for LN tolerance.

Thirty-eight percent out of 65 inbred lines analyzed for tryptophan content had optimum tryptophan contents although all the inbred lines were extracted from QPM populations. This is probably because early-generation selection for QPM alleles and desirable grain qualities was done using visual characterization under the light box and not through chemical analysis due to the large number of genotypes and the high cost of laboratory and molecular analysis. According to Vivek et al. [52], endosperm modification under the light box does not guarantee protein quality since the right dose of modifier genes is required for optimal amino acid accumulation. TZEEQI 414 had the highest tryptophan content of 0.094 and could serve as a good source of favorable genes for developing QPM populations and improving the yellow endosperm population.

The fifteen extra-early yellow inbred lines that had high positive base indices, but low tryptophan content levels could serve as good germplasm resource for developing LN tolerant normal endosperm hybrids. Furthermore, favorable tryptophan alleles could be introgressed into these inbred lines to increase the levels of tryptophan while maintaining LN tolerance to develop LN tolerant QPM lines. The four inbred lines TZEEIORQ 73A, TZEEQI 392, TZEEQI 394 and TZEEQI 408 that combined high positive base indices with high tryptophan levels could provide good sources of alleles for improving tryptophan contents of the orange and yellow source populations.

The genotype-by trait biplot (GT) analysis provides a graphical display of the inter-relationship among traits and helps to identify the best genotypes and traits for indirect selection. In this study, the GT analysis identified TZEEQI 394 and TZEEIORQ 73A as the best inbred lines for GY, PHT and EHT indicating that they possessed desirable genes for improving these traits and for hybrid production. The positioning of GY, PHT and EHT within the same sector containing the best two inbred lines TZEEQI 394 and TZEEIORQ 73A indicated that the traits were inter-related such that selection for tall plant and ear heights, and high EHT/PHT ratio could result in indirect selection for high GY. TZEEIORQ 4 was the best genotype for EPP and possessed favorable alleles for improving the trait although it was not very high yielding. Additionally, the results suggested that TZEEQI 368 may not have been the best genotype for improving earliness or for producing early maturing hybrids because it was late in maturity and possessed high ASI.

Due to the low heritability of GY under stress, high selection efficiency in LN breeding programs can be achieved through indirect selection with secondary traits that are easy to measure, have high heritability and strong correlation with grain yield [53]. In the present study, ASI and EPP were significantly associated with GY. However, due to the low heritability estimates of ASI and EPP, the traits may not be reliable for indirect selection for high grain yield. Indirect selection with secondary trait under stress only becomes more efficient than direct selection with GY when the secondary traits are highly heritable and strongly correlated with grain yield [54]. The other secondary traits PHT, EHT, EPP, EA and DS had significant and positive correlation with GY. The heritability estimates of the traits were also moderate to high. Therefore, selection for high GY with these traits would be possible. Contrarily, since DA, STYG, PA and HC were not significantly associated with GY, selection cannot be based on these traits. The results of our study corroborated the findings of Ziyomo and Bernado [54] who reported significant association between plant height and grain yield under stress. The authors also reported high selection efficiency and correlation of ASI with GY which was not consistent with our results.

Knowledge of the genetic diversity among inbred lines or within a population is important in selecting parental lines for maximizing heterosis without making all possible crosses among a set of inbred lines. Genetic diversity studies have been used to determine the phylogeny and genetic distances among lines extracted from the same source population for the purpose of initiating a hybrid program [4,55]. The average PIC (0.290) observed in the present study, representing the mean allelic diversity at a locus, was higher than the 0.19, 0.218 and 0.239 reported by [5,10,56], but similar to the 0.291 and 0.289 reported by [57,58], respectively. The average MAF was 0.275, with 3.0% of the SNPs showing MAF<0.1 and 12.1% having almost equal MAF (0.5±0.05) for the two alternative alleles. These values were found to be higher than those reported in other IITA and CIMMYT lines as reported by [5,10] who had MAF<0.1 and mean MAF of 0.16 and 0.202, respectively. Gene diversity of 0.363 observed in this study was higher than the 0.22 and 0.25 reported among IITA inbred lines by [5,10] but lower than the 0.390 reported by [59] respectively. Similar genetic diversity was reported by [60] among 70 IITA early provitamin A-QPM inbred population with 8170 DArTseq markers. The results corroborated the findings of [58,61] which concluded that tropical germplasm was highly diverse, usually exhibiting diversity of above 0.3. The differences between our results and results of other studies could be attributed to genotypic differences, number of genotypes used, number of markers and the genotyping platform used. In the present study, we used the SeqSNP targeted genotyping platform and selected markers with higher MAF based on previous studies, hence the high MAF values obtained were not surprising.

Genetic distance is a useful parameter for estimating the genetic similarity among individuals in a population [58]. In this study, there were wide average genetic distance (0.508) among the inbreds. Similarly, the pairwise genetic distance of majority of the inbred lines were high. Our results are comparable to those of [5] involving 96 IITA maize inbred lines using 15,047 SNP markers. Our findings suggested that most of the inbred lines studied were distinct and possessed unique alleles that could be invaluable to breeding programs in the tropics. The wide genetic distances observed in the inbred lines in the present study indicated that heterosis among the inbred lines could be high when used in hybrid breeding programs.

The average heterozygosity of 7% observed among the inbred lines exceeded the 3.34%, 3.8% and 5.6%, reported by [10,60,62], respectively but lower than the 8.6% reported by [63]. Similar result of 7% residual heterozygosity was reported by [5]. The relatively low heterozygosity observed for the inbred lines suggested that majority (93%) of the gene loci were fixed as expected of homozygous inbred lines. This was expected because most of the inbred lines were advanced generations (S6—S8) and were considered fixed for hybrid development (Badu-Apraku, 2019; Personal communication).

In the cluster analysis, majority of the lines were grouped based on the endosperm color, pedigree, and selection history. Even though most of the markers selected for this study were located within LN tolerance genomic regions, the pattern of clustering was not based on the performance under LN since both tolerant and susceptible lines were grouped together. Furthermore, no tryptophan content-based clustering pattern was observed. The failure of some lines to cluster based on selection history corroborated the findings of [64] who reported that inbred lines extracted from the same population may not share similar selection history and may not cluster together. Also, the absence of clustering based on LN tolerance or tryptophan content is supported by previous finding of [65,66] who reported that clustering among a set of CIMMYT inbreds and open pollinated varieties was independent of the phenotype or environmental adaptations. The high genetic distances between inbreds from different source populations which caused them to be grouped under different clusters suggested that hybrid crosses between yellow and orange lines from different clusters would produce higher heterosis compared to crosses between only yellow or orange lines within to the same cluster [6].

Population structure analysis also grouped the lines into five sub-populations largely based on pedigree and selection history. The F_ST_ values observed among the five sub-populations in the in the present study varied between 0.29 and 0.72 and were high and based on the interpretation by [67] which considered F_ST_ value of above 0.15 as significantly different populations. The F_ST_ values were similar to those previously reported in other tropical inbred lines by [5,68], of 0.33 and 0.93, respectively. The high F_ST_ values indicated that the inbred lines from the different groups possessed unique alleles that could be beneficial for improving LN populations in tropical breeding programs. In this study, the grouping of the lines under five sub-groups were different between the population structure and the phylogenic tree. This is attributable to the differences in the models and clustering algorithms used by the two methods. Inconsistent grouping pattern by different clustering methods have been reported by [10,12,69].

## Conclusions

The inbred lines exhibited wide pairwise genetic distances and expressed varying degrees of LN tolerance. The admixture model-based population structure and the unweighted pair group method with arithmetic mean placed the inbred lines into five groups each. The clustering patterns were neither according to tryptophan nor LN tolerance level, but were based on the endosperm color, pedigree, and selection history. Genetic distances were wider between the yellow and orange inbred lines compared to the distances between inbred lines of the same endosperm color. Most of the inbred lines selected for tryptophan analysis had low tryptophan content. TZEEIORQ 73A, TZEEQI 408, TZEEQI 392, TZEEQI 394 were LN tolerant and had optimal tryptophan contents. Fifteen of the inbred lines expressed LN tolerance but sub-optimal tryptophan contents. Inter-trait relationship existed between GY, PHT and EHT with TZEEQI 394 and TZEEIORQ 73A having high expressivity for these traits. The study indicated that the inbred lines are beneficial sources of alleles for QPM breeding programs for population improvement and developing LN tolerant varieties.

## Supporting information

S1 Table Description, cluster pattern and population structure of inbred lines. (XLSX)

S2 Table Soil chemical properties of experimental fields used in the study. (XLSX)

S3 Table Grain yield and other agronomic traits of inbred lines under low nitrogen. (XLSX)

S4 Table Summary statistics of the 1691 markers used to assess the diversity. (XLSX)

S5 Table Pairwise genetic distances among the inbred lines used in the study. (XLSX)
